# Hypertensive Heart Failure with Preserved Ejection Fraction: Guidelines vs. Randomized Controlled Trials Evidence Gaps

**DOI:** 10.3390/medicina62071222

**Published:** 2026-06-24

**Authors:** Georgios Mavraganis, Christos Fragoulis, Georgios Georgiopoulos, Kyriaki Mavromoustakou, Kyriakos Dimitriadis, Konstantinos Aznaouridis, Christina Chrysohoou, Kimon Stamatelopoulos, Konstantinos Tsioufis

**Affiliations:** 1 First Cardiology Clinic, School of Medicine, Hippokration General Hospital, National and Kapodistrian University of Athens, 11527 Athens, Greece; 2 Department of Clinical Therapeutics, School of Medicine, Alexandra Hospital, National and Kapodistrian University of Athens Medical School, 11527 Athens, Greece

**Keywords:** hypertension, heart failure, HFpEF, SGLT2 inhibitors, diastolic dysfunction, biomarkers, precision medicine, renal denervation

## Abstract

Hypertension is among the most important modifiable risk factors associated with heart failure with preserved ejection fraction (HFpEF) development and progression, yet guideline-directed blood pressure (BP) targets (<130/80 mmHg) and sodium–glucose co-transporter 2 inhibitor (SGLT2i) therapies lack dedicated randomized controlled trials (RCTs) in this specific group of patients. This narrative review synthesizes 2024 ESC/ESH and 2025 JSH meta-analyses, discussing the proposed pathophysiological framework linking hypertension-associated remodeling with HFpEF. Post hoc analyses from landmark trials (EMPEROR-Preserved, DELIVER) demonstrate consistent heart failure (HF) event reductions with SGLT2i (pooled HR 0.79, 95% CI 0.67–0.93), complemented by modest systolic BP lowering (−2.3 mmHg) and biomarker insights. Soluble ST2 and N-terminal pro-B-type natriuretic peptide (NT-proBNP) may contribute to risk stratification in HFpEF populations when interpreted in conjuction with imaging findings and clinical context; however, neither biomarker is specific for hypertension-mediated remodeling. Critical evidence gaps persist: heterogeneous BP thresholds across international guidelines, limited device therapy data (renal denervation showing −8.5 mmHg sustained reduction), and real-world implementation barriers among elderly/comorbid Europeans (adherence < 50%, polypharmacy risks). Hellenic HF Registry data highlight frailty prevalence (68% in patients > 75 years) complicating aggressive BP management. The review addresses phenotype-specific challenges through precision medicine approaches incorporating phenomapping and multi-biomarker panels (NRI 0.28 improvement). We advocate for dedicated HFpEF RCTs evaluating intensive vs. standard BP targets, SGLT2i sequencing with antihypertensives, and European real-world registries to bridge the translational gap. These strategies aim to transform guideline recommendations into optimized, patient-centered care for the rapidly expanding hypertensive HFpEF population.

## 1. Introduction

Heart failure with preserved ejection fraction (HFpEF) has become one of the fastest-growing cardiovascular (CV) syndromes worldwide and represents a major challenge for clinicians, researchers, and healthcare systems [1]. Unlike heart failure with reduced ejection fraction (HFrEF), which is characterized by impaired systolic function, HFpEF occurs when patients exhibit symptoms and signs of heart failure (HF) despite a preserved left ventricular ejection fraction (LVEF), typically ≥50% [1].

Hypertension is among the most important risk factors associated with the development of HFpEF [2]. Given the importance of hypertension in disease development, effective BP management is a key component of contemporary CV prevention strategies. Major international societies have issued recommendations aimed at reducing CV morbidity and mortality through tighter BP control. Guidelines from the European Society of Cardiology (ESC) and the European Society of Hypertension (ESH) generally recommend achieving systolic BP levels below 130 mmHg in patients at elevated CV risk when treatment is well tolerated [3]. Similar targets are endorsed by the American Heart Association and the American College of Cardiology [4]. Evidence supporting these recommendations demonstrates that intensive BP control reduces the incidence of CV events, including stroke, myocardial infarction, and HF hospitalization [5,6].

Despite these advances, uncertainty persists regarding the optimal BP targets specifically for patients with HFpEF. Most randomized controlled trials (RCTs) informing current guideline thresholds were conducted in populations with hypertension or general CV risk rather than cohorts diagnosed with HFpEF [5,7]. Consequently, BP targets for this population are largely extrapolated from broader studies instead of being derived from HFpEF-focused trials. This evidence gap complicates clinical decision-making for patients with established HFpEF.

At the same time, therapeutic strategies for HFpEF have evolved substantially in recent years [8,9,10]. For decades, treatment options were limited and most pharmacologic interventions failed to demonstrate significant improvements in clinical outcomes [11,12]. More recently, sodium–glucose co-transporter 2 inhibitors (SGLT2i) have transformed the therapeutic landscape of HF [8,9]. Originally developed for the treatment of type 2 diabetes mellitus, these agents have demonstrated substantial CV benefits independent of their metabolic effects [8,9]. Clinical trials show that SGLT2i reduce HF hospitalization and improve quality of life across the spectrum of HF phenotypes [8,13]. In addition to their primary CV benefits, SGLT2i produce modest reductions in systolic BP through mechanisms including osmotic diuresis and natriuresis, which may contribute to their overall therapeutic effects [14,15]. These benefits have been consistently demonstrated in HFpEF populations and have led to the rapid incorporation of SGLT2i into contemporary HF treatment guidelines [10,16,17].

Nevertheless, the role of SGLT2i within hypertension management strategies for HFpEF remains incompletely defined. Much of the available evidence comes from subgroup analyses rather than trials specifically designed to evaluate hypertensive HFpEF populations. Consequently, questions remain regarding the optimal sequencing of SGLT2i relative to traditional antihypertensive therapies. Although recent HFpEF trials have increased attention toward SGLT2i, other antihypertensive drug classes may also exert clinically relevant effects beyond BP lowering. Renin–angiotensin–aldosterone system (RAAS) inhibition through angiotensin-converting enzyme (ACE) inhibitors and angiotensin II receptor blockers (ARBs) has long been associated with attenuation of myocardial fibrosis, regression of left ventricular hypertrophy (LVH), endothelial protection, and vascular remodeling [18,19]. Similarly, mineralocorticoid receptor antagonists (MRAs) may influence extracellular matrix turnover and myocardial stiffness through antifibrotic mechanisms [20,21]. However, despite these biologically plausible effects, randomized evidence demonstrating definitive outcome modification in specific hypertensive HFpEF populations remains limited.

This narrative review examines the evolving literature at the intersection of hypertension and HFpEF, focusing on BP targets, emerging pharmacologic therapies, and the need for HFpEF-specific RCTs. By integrating evidence from recent guidelines and meta-analyses, the review aims to clarify current controversies and gaps as well as to outline future directions for research and clinical practice in the management of hypertensive HFpEF. Hence, the aim of this review is to critically examine the evidence gaps underlying current management of hypertension-associated HfpEF. Rather than advocating a single drug class, this review aims to examine whether future hypertension and HFpEF research should move beyond conventional BP-centric paradigms toward more phenotype-oriented approaches incorporating myocardial remodeling, fibrosis, inflammation, vascular dysfunction, and individualized therapeutic mechanisms.

## 2. Pathophysiology

### 2.1. Pathophysiology and Evidence Base

Building on the strong epidemiological association described above, hypertension represents the most important modifiable contributor to HFpEF development [22]. Epidemiological studies consistently demonstrate that a large proportion of patients with HFpEF have a history of long-standing hypertension, suggesting that chronic pressure overload plays a central role in disease development [2,23]. Recent guideline updates and meta-analyses have further clarified the mechanistic pathways linking hypertension to HFpEF and emphasized the importance of effective BP control alongside emerging pharmacologic therapies [3,10]. Persistent elevations in systemic BP are thought to contribute to chronic hemodynamic stress on the myocardium and vasculature, potentially promoting structural and functional alterations associated with HFpEF development [24,25]. However, much of the available evidence remains associative and mechanistic rather than derived from direct longitudinal demonstration of a uniform causal progression. Notably, large cohort analyses have demonstrated that hypertensive individuals have a substantially higher likelihood of developing HF compared with normotensive populations [26,27,28]. More precisely, meta-analytic data suggest that hypertension is associated with approximately an 80% increase in HFpEF risk, underscoring its central role in disease pathogenesis [29].

The relationship between hypertension and HFpEF involves both cardiac and systemic mechanisms. Chronic pressure overload promotes structural myocardial remodeling that progressively impairs ventricular relaxation and diastolic filling, both hallmarks of HFpEF [2,30]. In addition to direct myocardial effects, hypertension contributes to vascular abnormalities such as endothelial dysfunction, arterial stiffening, and microvascular impairment, all of which further exacerbate ventricular dysfunction [2,31].

### 2.2. Hypertension, Left Ventricular Hypertrophy, and Diastolic Dysfunction

The pathophysiological relationship between hypertension and HFpEF is primarily mediated through structural remodeling of the left ventricle [32]. Sustained elevations in arterial pressure increase ventricular afterload, stimulating adaptive myocardial hypertrophy aimed at maintaining cardiac output [2,26,33]. Over time, however, this compensatory response becomes maladaptive, resulting in increased myocardial stiffness, impaired relaxation, and ultimately diastolic dysfunction [2].

**Figure 1:**
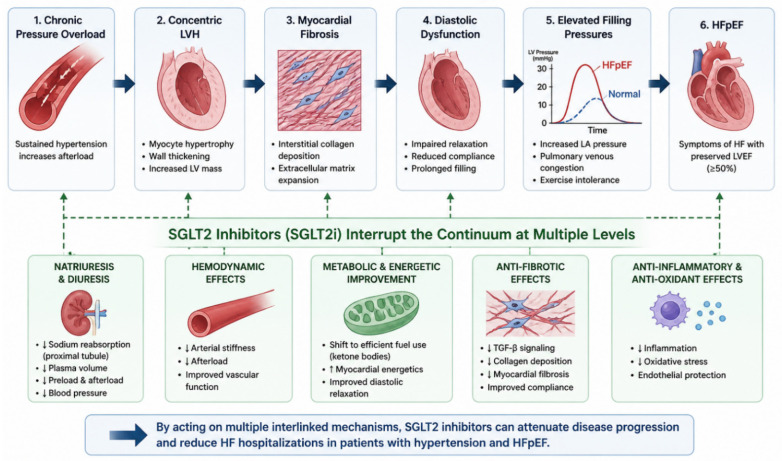
Proposed pathophysiological model linking hypertension-associated remodeling to HFpEF and potential multilevel effects of SGLT2i. The illustration represents a conceptual and proposed pathophysiological framework based on currently available evidence rather than a definitively validated longitudinal disease trajectory. Abbreviations: HFpEF, heart failure with preserved ejection fraction; HTN, hypertension; LVH, left ventricular hypertrophy; LA, left atrial; LVEF, left ventricular ejection fraction; SGLT2i, sodium–glucose co-transporter 2 inhibitor; HF, heart failure.

LVH represents a key intermediate stage in the transition from hypertension to HFpEF [26]. Chronic pressure overload promotes cardiomyocyte growth and extracellular matrix remodeling, particularly through increased collagen deposition and myocardial fibrosis, ultimately contributing to elevated filling pressures, a hallmark of HFpEF [32,34,35]. The proposed pathophysiological framework linking hypertension-associated remodeling to HFpEF and the multilevel effects of SGLT2i is summarized in Figure 1.

Hypertension also contributes to abnormalities in coronary microcirculation [36]. Endothelial dysfunction and microvascular rarefaction reduce myocardial perfusion reserve, further aggravating ventricular stiffness and impaired relaxation [37]. Chronic inflammation and oxidative stress amplify these processes by promoting progressive myocardial fibrosis and ventricular stiffening [2].

Importantly, hypertension-related remodeling is not limited to the left ventricle. Structural and functional changes also occur in the left atrium and pulmonary vasculature, contributing to atrial enlargement, atrial fibrillation, and pulmonary hypertension—conditions that frequently coexist in HFpEF populations [38,39]. These findings highlight the multisystem nature of the disease.

### 2.3. BP Targets and Guideline Recommendations

Since hypertension plays a central role in HFpEF development, optimal BP control has emerged as a key preventive and therapeutic strategy. Recent updates from major CV societies emphasize stricter BP targets in patients at risk for CV disease. Current ESC recommendations advocate achieving guideline-recommended BP targets in patients with hypertension, particularly those with evidence of structural heart disease [3].

Large observational studies and RCTs support these recommendations, demonstrating that intensive BP lowering is associated with reductions in CV events, including HF hospitalization [40,41]. Effective BP control can also attenuate ventricular remodeling, reduce myocardial stiffness, and slow the progression of diastolic dysfunction [42,43].

Emerging evidence further suggests that early BP management may prevent the transition from asymptomatic diastolic dysfunction to clinically overt HFpEF [44,45]. This concept aligns with broader strategies for HF prevention that emphasize early intervention before irreversible structural damage occurs.

Despite these advances, achieving optimal BP control in HFpEF populations remains challenging. Many patients present with multiple comorbidities—including obesity, chronic kidney disease (CKD), and metabolic syndrome—that complicate hypertension management [46]. Consequently, multidimensional therapeutic strategies are often required. A comparison of major society guidelines highlights both consensus and areas of divergence in the management of hypertension-associated HFpEF (Table 1).

### 2.4. SGLT2i and CV Benefits

SGLT2i have recently emerged as a major therapeutic advance in the management of HF. Although initially developed for diabetes treatment, these agents have demonstrated robust CV benefits that extend beyond glucose control [8,9].

Large RCTs evaluating SGLT2i in patients with HFpEF have reported significant reductions in HF hospitalization and CV mortality [8,9]. Meta-analyses indicate that treatment with these agents is associated with over 20% relative reduction in HF events [47,48]. Notably, these benefits appear consistent across patient populations, including individuals with and without diabetes [47].

The cardioprotective effects of SGLT2i are multifactorial. They promote osmotic diuresis and natriuresis, leading to reductions in plasma volume and ventricular preload [49]. Additional benefits include improvements in vascular function, reductions in systemic BP, and enhanced myocardial energetics through shifts in cardiac metabolism toward more efficient substrates such as ketone bodies [50].

Experimental studies also suggest that these agents may directly influence myocardial remodeling by reducing inflammation, oxidative stress, and myocardial fibrosis—processes central to the development of diastolic dysfunction [51,52]. Improvements in renal function and reductions in interstitial fluid accumulation may further contribute to CV stability [15,53].

From a hypertension perspective, SGLT2i produce modest reductions in systolic BP, complementing the effects of conventional antihypertensive therapies [15]. Although this effect alone does not fully explain their CV benefits, it may complement conventional antihypertensive therapies by improving hemodynamic stability and reducing cardiac workload.

Despite their favorable cardiovascular profile, SGLT2i are associated with several clinically relevant adverse effects that may complicate use in elderly and multimorbid HFpEF populations. Increased urinary frequency, osmotic diuresis, genital and urinary tract infections, volume depletion, and symptomatic hypotension may limit tolerability in selected patients [17,54,55]. In elderly individuals, particularly those with frailty, mobility limitations, urinary incontinence, or prostatic hypertrophy, these effects may significantly influence adherence and quality of life. Careful patient selection and individualized monitoring therefore remain essential.

### 2.5. RAAS Inhibition and Antifibrotic Strategies

RAAS activation plays a central role in hypertensive cardiac remodeling through promotion of myocardial fibrosis, collagen deposition, endothelial dysfunction, and left ventricular hypertrophy [56,57]. Consequently, ACE inhibitors and ARBs may exert CVeffects beyond BP lowering, including mitigation of adverse cardiac remodeling, regression of LVH, and modulation of profibrotic signaling pathways involved in myocardial structural remodeling [19,58]. Although major HFpEF trials involving RAAS modulation have yielded mixed outcome results, these agents remain mechanistically relevant in hypertension-associated remodeling phenotypes. Nevertheless, definitive randomized evidence demonstrating outcome modification in hypertensive HFpEF populations remains limited.

### 2.6. Mineralocorticoid Receptor Antagonists

MRAs, including spironolactone and eplerenone, target key mechanisms in hypertensive HFpEF by inhibiting aldosterone-mediated sodium retention, inflammation, and myocardial fibrosis [59,60]. These effects may improve ventricular stiffness and diastolic function while providing modest BP reduction, particularly in resistant hypertension [61]. Evidence from the TOPCAT trial suggests a reduction in HF hospitalizations, although effects on mortality remain uncertain, leading to a cautious role in current guidelines [12]. Although spironolactone demonstrated reductions in HF hospitalization in selected HFpEF populations, interpretation was complicated by regional heterogeneity and variable adherence [62]. However, MRAs remain of interest in hypertensive HFpEF given their potential antifibrotic effects and ability to influence extracellular matrix remodeling and myocardial stiffness [20,21]. Moreover, MRAs reduce BP by blocking aldosterone-mediated sodium retention and vascular remodeling, with randomized evidence—e.g., PATHWAY-2 trial—demonstrating that spironolactone is superior to other add-on therapies in resistant hypertension [5]. Nevertheless, their use requires careful monitoring due to risks of hyperkalemia and renal dysfunction, especially in patients with CKD [63,64]. Overall, MRAs may offer complementary benefits alongside standard antihypertensive therapy and SGLT2i, particularly in phenotypes characterized by LVH and fibrosis, although further targeted trials are needed.

### 2.7. Integrating Hypertension Control and Novel Therapies in HFpEF Management

The integration of effective BP management with emerging pharmacologic therapies represents a central component of modern HFpEF care. While traditional antihypertensive agents remain essential for controlling BP and preventing structural cardiac changes, novel treatments such as SGLT2i provide additional mechanisms that target underlying disease processes.

Current evidence suggests that combining optimized BP control with SGLT2i therapy may offer complementary benefits [8,9]. By reducing afterload, improving myocardial relaxation, and alleviating systemic congestion, such strategies may help slow disease progression and reduce clinical events [49].

As research continues to evolve, future studies are expected to clarify the optimal sequencing and combination of therapies for patients with hypertension who are at risk of developing HFpEF. As shown in Table 2, the heterogeneity of trial outcomes and the modest reductions in systolic BP underscore the complex pathophysiology of HFpEF and the limited role of BP lowering alone. Advances in biomarker profiling, imaging techniques, and precision medicine may also enable earlier identification of high-risk individuals and more personalized treatment strategies [65,66].

In summary, hypertension is strongly associated with HFpEF development and progression through mechanisms involving LVH, myocardial fibrosis, and impaired diastolic function. Contemporary evidence supports intensive BP control and highlights the transformative role of SGLT2i in reducing HF events. Together, these insights provide a strong foundation for improved prevention and management strategies in patients with hypertensive heart disease.

## 3. Guideline Gaps and Unresolved Questions in Hypertension-Driven HFpEF

Despite substantial advances in understanding the relationship between hypertension and HFpEF, important gaps remain in current clinical guidelines and the supporting evidence base. Although hypertension is widely recognized as the dominant modifiable risk factor for HFpEF, recommendations for BP management in this population are largely extrapolated from broader CV studies rather than trials specifically designed for HFpEF (Table 3) [68,69].

### 3.1. Uncertainty Around Optimal BP Targets

One of the most significant unresolved questions concerns the optimal BP targets for patients with hypertensive heart disease who are at risk for or already diagnosed with HFpEF. Current international guidelines generally recommend tight BP control in patients with elevated CV risk [3,4]. However, these targets are based largely on trials evaluating general CV outcomes—such as myocardial infarction, stroke, or overall HF incidence—rather than studies specifically focused on HFpEF populations [5,7]. Consequently, the BP targets required to prevent or manage HFpEF may differ from those established for broader CV risk reduction.

Recent meta-analyses attempting to address this question have produced heterogeneous findings. Some studies suggest that intensive BP control is associated with reductions in HF events and improvements in cardiac remodeling [5,73]. Others indicate that overly aggressive BP lowering may provide benefits in certain subgroups although it could increase the risk of adverse outcomes such as hypotension, renal dysfunction, electrolyte abnormalities or impaired coronary perfusion, especially in elderly patients [5,74].

These inconsistencies are reflected in subtle differences among international guidelines. For example, both European and Japanese hypertension guidelines broadly support BP targets below 130/80 mmHg, but their interpretations of the underlying evidence differ [3,4,75]. Some emphasize prevention of structural cardiac remodeling, whereas others focus on general CV risk reduction rather than HFpEF-specific outcomes [5,76].

The lack of consensus largely reflects the absence of RCTs specifically designed to test BP targets in patients with established or at high-risk for HFpEF. Most available data originate from observational studies or secondary analyses of hypertension trials not intended to evaluate this population. As a result, current recommendations rely heavily on indirect evidence.

Another challenge arises from the heterogeneity of HFpEF itself. The syndrome encompasses multiple phenotypes characterized by varying contributions of hypertension, obesity, systemic inflammation, and myocardial remodeling [2,24]. It is therefore possible that optimal BP targets differ across patient subgroups. For example, individuals with obesity-related HFpEF may require different hemodynamic goals compared with those whose disease is primarily driven by long-standing hypertensive cardiac remodeling [66,77,78].

Without adequately powered, phenotype-specific trials, BP targets for HFpEF remain generalized and may not fully reflect the diverse clinical presentations of the syndrome.

### 3.2. Limitations of Current Evidence for SGLT2i in Hypertensive HFpEF

Another area of uncertainty involves the role of SGLT2i in patients with hypertensive HFpEF. These agents have rapidly become central to HF management after multiple trials demonstrated reductions in hospitalization and CV events across both preserved and reduced LVEF populations [8,9,13]. Consequently, contemporary guidelines now recommend SGLT2i as part of standard therapy for HFpEF [17].

However, most supporting evidence comes from large trials enrolling heterogeneous HF populations with diverse underlying etiologies. Although EMPEROR-Preserved and DELIVER demonstrated significant reductions in HF hospitalization among trial-defined HFpEF populations, these studies were not specifically designed to validate a distinct hypertension-mediated HFpEF phenotype [8,9]. Consequently, current evidence supports the use of SGLT2i in broad HFpEF populations; however, it does not conclusively demonstrate reversal of hypertension-associated myocardial fibrosis or direct modification of a defined hypertension-mediated cardiomyopathic substrate [24]. Although hypertension was highly prevalent among participants, these studies were not specifically designed to investigate hypertensive HFpEF as a distinct clinical phenotype.

Subgroup and post hoc analyses suggest that SGLT2i provide consistent benefits across patients with and without diabetes and across different baseline BP levels [79,80]. While encouraging, these findings do not fully clarify whether the therapeutic effects differ in hypertension-driven HFpEF.

This distinction is important because hypertensive HFpEF is characterized by specific structural and hemodynamic abnormalities, including concentric LVH, increased arterial stiffness, and pronounced diastolic dysfunction [31,81]. Proposed antifibrotic and remodeling effects of SGLT2i remain largely mechanistic or exploratory and require confirmation in phenotype-specific studies [82,83]. It remains uncertain whether SGLT2i directly modify these structural processes or whether their benefits primarily arise from systemic effects such as natriuresis, improved renal function, and reductions in circulating volume.

Their modest BP-lowering effect also raises questions about their role within hypertension management. Although these agents typically reduce systolic BP by several millimeters of mercury [84], this reduction alone is unlikely to fully account for their observed CV benefits [85]. Whether SGLT2i should be viewed primarily as adjunct antihypertensive agents, HF therapies, or metabolic modulators remains an area of active investigation. SGLT2i should therefore be viewed within the broader context of multidimensional cardiovascular remodeling therapies rather than as isolated or uniquely privileged interventions.

Another unresolved issue involves the timing of therapy initiation. Most trials enrolled patients with established symptomatic HFpEF [8,9], leaving unanswered questions about whether earlier use could prevent progression from asymptomatic diastolic dysfunction to overt HF [86].

Prospective trials examining SGLT2i in patients with hypertensive cardiac remodeling prior to the onset of symptomatic HFpEF could therefore provide important insights into disease prevention strategies. In patients with persistent hypertension despite optimized pharmacologic therapy, alternative non-pharmacologic strategies have begun to emerge, including device-based interventions targeting neurohormonal and hemodynamic pathways.

### 3.3. Device Therapies in Resistant Hypertensive HFpEF

Device-based interventions address limitations of pharmacologic therapy in resistant hypertension complicating HFpEF. Renal denervation (RDN) reduces sympathetic outflow, achieving sustained systolic BP reductions (−8.5 mmHg at 24 months vs. sham) in the SPYRAL HTN-ON MED trial [87]. Post hoc analyses suggest RDN attenuates LVH regression in hypertensive HFpEF, warranting dedicated trials [88,89].

Baroreflex activation therapy and central iliac arteriovenous anastomosis represent emerging options for patients with symptomatic HFpEF and uncontrolled hypertension despite ≥ 3 agents. Cardiovascular implantable electronic devices (CIEDs) like implantable cardioverter-defibrillator/cardiac resynchronization therapy pacemaker show limited mortality benefit in HFpEF, but His-bundle pacing may improve diastolic synchrony in hypertensive cohorts with conduction abnormalities [90].

European real-world data from ARIC/Swedish HF registries and Hellenic studies (frailty 68% in HFpEF > 75 years) [91,92] will clarify device utility in multimorbid HFpEF phenotypes.

### 3.4. The Need for Phenotype-Specific Trials

These limitations highlight a broader challenge in HFpEF research: the need for phenotype-specific clinical trials. Unlike HFrEF, HFpEF represents a heterogeneous group of conditions with diverse underlying mechanisms. Hypertension-driven HFpEF is among the most common phenotypes, yet it remains insufficiently studied as a distinct entity.

Future trials specifically targeting hypertensive HFpEF could address several key questions. First, they could evaluate whether intensive BP targets improve diastolic function, ventricular remodeling, or clinical outcomes. Second, they could clarify the extent to which SGLT2i influence the structural and metabolic pathways involved in hypertensive cardiac remodeling. Finally, these studies could explore whether combination therapies targeting both systemic hypertension and myocardial fibrosis provide additive or synergistic benefits.

Advances in imaging, biomarker profiling, and machine learning-based phenotyping may facilitate the identification of more homogeneous patient populations for such trials. Improved patient selection and study design could generate more precise evidence to guide therapeutic decisions in hypertensive HFpEF.

### 3.5. Unresolved Questions

Importantly, alternative conceptual perspectives regarding hypertensive heart disease and HFpEF pathophysiology continue to evolve. While hypertension-induced structural changes—such as concentric LVH and myocardial fibrosis—are commonly implicated in the development of HFpEF [93], longitudinal studies establishing isolated hypertensive heart disease as a causally sufficient and inherently progressive cardiomyopathy remain scarce. Accordingly, epidemiological associations between hypertension and HFpEF should not be interpreted as definitive proof of a direct causal disease continuum, particularly given the marked phenotypic heterogeneity of contemporary HFpEF populations [24]. Contemporary HFpEF populations are highly heterogeneous and are frequently defined using syndromic clinical criteria, natriuretic peptide thresholds, and comorbidity profiles rather than direct validation of a specific hypertension-mediated myocardial disease phenotype [94]. Recent reappraisal of historical, imaging, histopathological, and clinical trial evidence has highlighted these ongoing uncertainties and the need for more precise mechanistic phenotyping in future HFpEF research [95].

### 3.6. Implications for Future Guidelines

Until such data becomes available, clinical guidelines will continue to rely on extrapolation from broader CV and HF studies. While these recommendations provide valuable guidance, they inevitably involve a degree of uncertainty when applied to specific HFpEF phenotypes.

Clinicians should therefore recognize that current BP targets and pharmacologic strategies represent the best available evidence but may not fully capture disease-specific mechanisms in hypertensive HFpEF. At the same time, guideline committees and research organizations should prioritize trials designed to address these unresolved questions.

In summary, although hypertension is widely acknowledged as a central driver of HFpEF, further investigation is warranted. Optimal BP targets have not been definitively established through dedicated RCTs, and the benefits of SGLT2i in hypertension-driven HFpEF are largely supported by secondary analyses rather than prospective studies. Addressing these gaps will be essential for refining treatment strategies and improving outcomes in this rapidly growing patient population.

Despite growing recognition of hypertension as a central mechanistic driver of HFpEF, substantial discrepancies persist between contemporary guideline recommendations and the availability of dedicated randomized evidence. Current management strategies largely rely on extrapolation from broader hypertension or HF populations rather than trials specifically designed for hypertensive HFpEF phenotypes. As summarized in Table 4, several key therapeutic domains—including BP targets, SGLT2i use, biomarker-guided management, and device-based therapies—remain supported primarily by post hoc analyses, observational data, or expert consensus. These limitations highlight the urgent need for RCTs capable of defining optimal therapeutic strategies and refining future guideline recommendations in hypertensive HFpEF.

## 4. Real-World Barriers to Optimal Management of Hypertension in HFpEF

Although contemporary guidelines emphasize aggressive BP control and the use of emerging pharmacologic therapies in HFpEF, translating these recommendations into routine clinical practice remains challenging. Patients with HFpEF are typically older and frequently present with multiple comorbidities, functional limitations, and complex medication regimens [96,97,98]. These factors contribute to suboptimal adherence, therapeutic inertia, and increased risk of adverse effects from intensive pharmacologic therapy [99]. Consequently, a substantial gap persists between guideline recommendations and real-world care.

Three major barriers consistently emerge: the advanced age and comorbidity burden of HFpEF populations, the challenges associated with polypharmacy and potential BP over-lowering, and the absence of disease-specific treatment algorithms tailored to hypertensive HFpEF [2,33].

### 4.1. Aging and Multimorbidity

HFpEF predominantly affects older adults, with many patients diagnosed in their seventh or eighth decade of life [17]. Aging is associated with physiological changes that complicate hypertension management, including increased arterial stiffness, reduced renal function, and altered autonomic regulation [100,101,102]. These changes contribute to greater BP variability and increased susceptibility to hypotension, as well as to adverse CV events when antihypertensive therapy is intensified [103]. In addition, HFpEF is frequently accompanied by multiple comorbid conditions such as CKD, atrial fibrillation, diabetes mellitus, obesity, and frailty [33]. Each of these conditions may influence therapeutic decisions and limit the feasibility of aggressive BP-lowering strategies.

CKD represents a particularly important challenge. Many antihypertensive medications—including renin–angiotensin system inhibitors and diuretics—require cautious use in patients with impaired renal function [104,105]. Excessive BP reduction may compromise renal perfusion and accelerate kidney function decline, forcing clinicians to balance CV benefits against potential renal risks [5].

Frailty further complicates treatment decisions. Elderly Europeans face particular challenges in HFpEF management. Among 38,843 older HF patients (mean age 80.4 years), 68.3% exhibited frailty—47.5% mild, 20.8% severe—with frail patients experiencing double the readmissions (median 2 vs. 1; interquartile range 3 vs. 2) compared to non-frail individuals [106]. In hypertensive elderly cohorts, frailty independently predicts poor adherence (<45% for ≥3 antihypertensives), driven by cognitive decline and polypharmacy [107,108]. Patients with reduced physiological reserve may be particularly vulnerable to adverse drug effects, including orthostatic hypotension, dizziness, and falls [109,110,111]. In such individuals, clinicians often adopt more conservative BP targets despite guideline recommendations. Medication adherence is another major obstacle. Cognitive impairment, complex treatment regimens, and limited social support frequently contribute to poor adherence among elderly patients with multiple CV medications [112,113,114]. Observational studies report low adherence rates in patients receiving complex antihypertensive therapy [113].

Socioeconomic and healthcare access factors may further limit effective BP control [113]. Frequent monitoring, medication adjustments, and follow-up visits are often required but may not always be feasible for elderly patients.

### 4.2. Polypharmacy and BP Over-Lowering

Polypharmacy is another defining feature of HFpEF populations. Due to their multiple chronic conditions patients frequently receive numerous medications including antihypertensive agents, diuretics, anticoagulants, lipid-lowering drugs, and glucose-lowering therapies [115,116]. This cumulative medication burden increases the risk of drug interactions, adverse effects, and treatment discontinuation.

In hypertension management, polypharmacy may lead to unintended BP over-reduction, particularly when multiple agents with overlapping hemodynamic effects are used simultaneously [5]. Combinations of diuretics, renin–angiotensin system inhibitors, beta-blockers, and novel HF therapies may produce substantial BP reductions that compromise organ perfusion.

These risks are particularly pronounced in elderly HFpEF patients, whose CV systems may have limited adaptive capacity. Reduced baroreceptor sensitivity and impaired autonomic responses make it more difficult to maintain stable BP during aggressive therapy [117,118]. Orthostatic hypotension, a common consequence of excessive BP lowering, significantly increases fall risk and associated complications [117,119].

Another challenge involves therapeutic prioritization. Clinicians must determine which medications offer the greatest clinical benefit while minimizing potential harms [120]. The absence of clear prioritization frameworks in current guidelines contributes to variability in clinical practice.

The introduction of agents such as SGLT2i further complicates medication regimens. Although these drugs provide important CV benefits, their addition may require adjustments to other medications to prevent hypotension or volume depletion [121].

### 4.3. Lack of Tailored Implementation Strategies

A third barrier involves the absence of treatment algorithms specifically designed for hypertensive HFpEF. Hypertension guidelines provide general recommendations for BP control, but they do not always address the unique pathophysiological characteristics of HFpEF [3,4,75]. Conversely, HF guidelines often emphasize pharmacologic therapy without detailed guidance on BP optimization in hypertensive cardiac remodeling [10,17].

This gap creates uncertainty for clinicians managing patients with overlapping CV conditions. For example, the optimal sequence of therapies for individuals with both hypertension and HFpEF remains poorly defined. It is unclear whether treatment strategies should prioritize specific drug classes, initiate combination therapy earlier, or adjust intensity across different HFpEF phenotypes.

Another challenge involves identifying patients who would benefit most from intensive BP management. Because HFpEF encompasses multiple phenotypes characterized by varying degrees of structural heart disease, metabolic dysfunction, and systemic inflammation, clinicians may struggle to determine which patients require aggressive intervention.

Healthcare system factors also contribute to management challenges. Fragmentation of care among cardiology, nephrology, and primary care providers may lead to inconsistent treatment strategies and conflicting priorities. Improved coordination among healthcare professionals is therefore essential for effective implementation of guideline-directed therapy [122,123].

Digital health technologies may offer potential solutions. Home BP monitoring, telemedicine consultations, and automated medication reminders could improve adherence and allow more precise BP management [124,125]. However, integration of these tools into routine practice remains uneven and may be limited by cost and accessibility [126].

### 4.4. Bridging the Evidence–Practice Gap

Addressing real-world barriers to hypertension management in HFpEF requires a multifaceted approach combining clinical research, guideline refinement, and healthcare system improvements. Future research should focus on developing practical treatment algorithms tailored to hypertensive HFpEF, particularly for elderly and multimorbid populations.

Simplifying medication regimens, strengthening patient education, and improving coordination among healthcare providers may reduce the burden of polypharmacy and improve adherence. Personalized strategies that consider frailty, comorbidity burden, and functional status may also help balance the benefits of BP control against the risks of intensive therapy.

Ultimately, bridging the gap between guideline recommendations and real-world practice will be critical for improving outcomes in patients with hypertensive HFpEF.

## 5. Future Directions

Despite recent advances in the management of hypertension and HFpEF, further research should focus on optimal BP targets and the integration of novel therapies such as SGLT2i. As discussed before, most current recommendations are extrapolated from broader CV studies rather than trials specifically designed for hypertensive HFpEF. Addressing these limitations will require targeted RCTs and precision-medicine approaches.

### 5.1. Need for Dedicated Randomized Trials

One major limitation is the absence of RCTs designed to determine optimal BP management specifically in HFpEF. Although current guidelines recommend BP levels below 130/80 mmHg, these targets are based on general CV populations rather than patients with established HFpEF.

Future trials comparing different BP targets—such as <130/80 mmHg versus <140/90 mmHg—in hypertensive HFpEF populations could provide valuable insights. Relevant outcomes should include HF hospitalization, exercise capacity, and changes in ventricular structure or function.

Another important research area involves evaluating combination strategies incorporating SGLT2i alongside antihypertensive therapy. Although these agents reduce HF events, most available data derive from secondary analyses. Dedicated studies could clarify optimal treatment sequencing, timing of therapy initiation, and potential synergistic effects between SGLT2i and conventional antihypertensive medications.

Ensuring adequate representation of elderly patients, women, and individuals with comorbidities will be essential, as these groups are frequently underrepresented in CV trials but constitute a large proportion of the HFpEF population [127,128].

### 5.2. Precision Medicine and Biomarkers

Precision-medicine approaches offer another promising direction for improving HFpEF management. Because HFpEF encompasses heterogeneous phenotypes—including hypertensive, metabolic, and inflammatory forms—a uniform treatment strategy is unlikely to be optimal [24].

Soluble ST2 and natriuretic peptides may contribute to risk stratification in HFpEF populations [129,130,131], although their interpretation requires integration with imaging findings and clinical context given the influence of age, atrial fibrillation, renal dysfunction, and other comorbidities [94,132]. Soluble ST2 (sST2), a marker of myocardial stress and fibrosis, has emerged as a promising candidate given its association with processes central to hypertensive HFpEF [133,134,135]. Elevated sST2 levels (>35 ng/mL) may identify early myocardial remodeling before the onset of symptomatic disease, potentially allowing earlier intervention.

Additional biomarkers such as N-terminal pro-B-type natriuretic peptide (NT-proBNP) and high-sensitivity cardiac troponin T may also contribute to improved risk stratification [136,137]. When combined with imaging modalities such as echocardiography or cardiac magnetic resonance imaging, biomarker-guided strategies could provide a more comprehensive assessment of cardiac structure and function. Although natriuretic peptides remain central to HF diagnostic and prognostic algorithms, NT-proBNP elevations are not specific for hypertensive HFpEF and may reflect multiple cardiac and systemic conditions including atrial fibrillation, renal dysfunction, pulmonary hypertension, and advanced age [94,129,130,131,132]. Consequently, biomarker interpretation should be integrated with structural imaging and comprehensive clinical assessment rather than used in isolation to infer progressive hypertensive remodeling.

### 5.3. Toward a Targeted Management Approach

Future strategies for hypertensive HFpEF should integrate evidence from dedicated clinical trials with biomarker-based risk stratification. Personalized approaches could help refine BP targets, optimize drug selection, and monitor therapeutic responses more effectively.

Future studies may benefit from distinguishing preventive and therapeutic trial frameworks. One approach would involve recruiting hypertensive patients with early structural remodeling (e.g., LVH, impaired diastolic relaxation, elevated fibrosis markers, or left atrial remodeling) before overt HFpEF develops, thereby assessing whether targeted intervention can prevent progression toward symptomatic disease. Complementary trials in established HFpEF populations should prioritize more homogeneous phenotypic stratification including hypertension-dominant remodeling phenotypes, in order to better evaluate differential therapeutic responses and mechanisms of action.

Combining intensive BP control with SGLT2i therapy in carefully selected high-risk patients may offer additive benefits, although confirmation in well-designed studies is required.

Overall, closing current evidence gaps will require both targeted RCTs and advances in precision medicine aimed at improving risk stratification and therapeutic decision-making.

## 6. Conclusions

Hypertension remains a central driver of HFpEF development and progression. Although current guidelines recommend aggressive BP control and increasingly endorse SGLT2i for HFpEF management, further research is warranted. Moreover, the evolving management of hypertension-associated HFpEF may ultimately require a broader therapeutic framework extending beyond BP reduction alone. Future therapeutic strategies should evaluate not only SGLT2i but also RAAS inhibition, MRAs, and combination approaches targeting fibrosis, inflammation, endothelial dysfunction and ventricular remodeling.

The absence of HFpEF-specific RCTs addressing BP targets and therapeutic strategies limits the ability of clinicians to make fully evidence-based decisions. Real-world challenges—including multimorbidity, polypharmacy, and poor adherence—further complicate implementation of guideline recommendations.

Future research should prioritize phenotype-specific clinical trials, biomarker-guided risk stratification, and pragmatic strategies aimed at translating evidence into clinical practice. Addressing these challenges will be critical for improving outcomes in the rapidly growing population of patients with hypertensive HFpEF.

## Figures and Tables

**Table 1:** Comparison of ESC and AHA/ACC recommendations in hypertension-associated HFpEF.

Clinical Domain	ESC Guidelines	AHA/ACC Guidelines
BP target	<130/80 mmHg if tolerated (Class I, Level C)	<130/80 mmHg (Class I, Level C-EO)
SGLT2i	Recommended in HFpEF (Class I, Level A)	Recommended in HFpEF (Class IIa, Level B-R)
MRAs	Considered in selected patients (Not applicable)	May be considered (Class IIb, Level B-R)
RAAS inhibition	Recommended for BP control/comorbidities (Not applicable for mortality, mainly used for comorbidities)	Recommended particularly with hypertension/comorbid disease (Class IIb, Level B-R)
Phenotype-specific guidance	Limited	Limited

Abbreviations: ESC, European Society of Cardiology, AHA, American Heart Association; ACC, American College of Cardiology; HFpEF, heart failure with preserved ejection fraction; BP, blood pressure; SGLT2i, sodium–glucose co-transporter 2 inhibitor; MRA, mineralocorticoid receptor antagonist; RAAS, renin–angiotensin–aldosterone system.

**Table 2:** Comparison of international blood pressure targets relevant to hypertensive HFpEF management.

Guideline	Publication Year	Target Population	SBP Target	DBP Target	HFpEF-Specific Evidence	Key Notes
ESC/ESH 2024 [3]	2024	High CV risk + HTN	<130 mmHg	<80 mmHg	Extrapolated	“If tolerated”; emphasizes LVH regression
AHA/ACC 2025 [4]	2025	General HTN (age ≥ 65)	<130 mmHg *	<80 mmHg	None	* Individualized in frailty/CKD
JSH 2025 [67]	2025	Elderly HTN (>75 years)	<140 mmHg	<90 mmHg	None	More conservative in advanced age
ACC/AHA HF 2022 [10]	2022	Established HFpEF	<130/80 mmHg **	<80 mmHg	Post hoc SGLT2i data	** Combined with GDMT; monitor orthostasis

* If tolerated without adverse effects. ** In context of SGLT2i/MRA therapy. Note: Current guidelines recommend intensive BP control for hypertensive HFpEF but rely on extrapolation from general cardiovascular risk populations rather than phenotype-specific randomized controlled trials. ESC/ESH targets prioritize structural remodeling prevention, while JSH adopts a more conservative approach for elderly patients. Abbreviations: ESC, European Society of Cardiology; ESH, European Society of Hypertension; AHA, American Heart Association; JSH, Japanese Society of Hypertension; ACC, American College of Cardiology; HF, heart failure; SBP, systolic blood pressure; DBP, diastolic blood pressure; CV, cardiovascular; HTN, hypertension; LVH, left ventricular hypertrophy; CKD, chronic kidney disease; HFpEF, heart failure with preserved ejection fraction; SGLT2i, sodium–glucose co-transporter 2 inhibitors; GDMT, guideline-directed medical therapy; MRA, mineralocorticoid receptor antagonist.

**Table 3:** Landmark HFpEF trials and associated blood pressure effects.

Trial	Year	N (HFpEF)	HTN Prevalence	Intervention	Primary Outcome HR/Effect Size (95% CI)	SBP Reduction
EMPEROR-Preserved [8]	2021	5988	90.6%	Empaglifozin vs. placebo	0.79 (0.69–0.90)	−2.0 mmHg
DELIVER [9]	2022	6263	88.7%	Dapaglifozin vs. placebo	0.82 (0.73–0.92)	−2.5 mmHg
PARAGON-HF [70]	2019	4822	95.1%	Sacubitril/valsartan vs. valsartan	0.87 (0.75–1.01)	−4.5 mmHg
TOPCAT [12]	2014	3445	91.3%	Spironolactone vs. placebo	0.89 (0.77–1.04)	−2.5 mmHg
PRESERVED-HF [71]	2021	324	~80%	Dapaglifozin vs. placebo	+5.8 (2.3–9.4) *	−0.6 mmHg
STEP-HFpEF [72]	2023	529	81.9%	Semaglutide vs. placebo	+7.8 (4.8–10.9) *	−2.9 mmHg
Meta-analysis [48]	2022–2024	8116	84%	Pooled SGLT2i vs. placebo	0.77 (0.67–0.88)	−1.8 mmHg

* Primary outcomes correspond to change in KCCQ-CSS while other outcomes are defined as a composite of cardiovascular death and/or HF hospitalization. Abbreviations: HFpEF, heart failure with preserved ejection fraction; HTN, hypertension; HR, hazard ratio; CI, confidence interval; SBP, systolic blood pressure; HF, heart failure; SGLT2i, sodium–glucose co-transporter 2 inhibitors; KCCQ, Kansas City Cardiomyopathy Questionnaire; CSS, Clinical Summary Score. Note: SBP change represents mean between-group difference derived from secondary or exploratory analyses; these trials were not primarily designed to evaluate blood pressure reduction.

**Table 4:** Guideline recommendations versus dedicated randomized evidence in hypertensive HFpEF.

Clinical Domain	Current Guideline Position	Dedicated HFpEF RCT Evidence	Main Supporting Studies	Major Evidence Gap	Future Research Priorities
BP targets	Recommend BP control, usually <130/80 mmHg if tolerated	No dedicated hypertensive HFpEF BP-target RCTs	SPRINT, ESC/ESH 2024, ACC/AHA HF 2022	Targets extrapolated from general hypertension/CV-risk trials	RCTs comparing intensive vs. standard BP targets in hypertensive HFpEF
SGLT2 inhibitors	Recommended for HFpEF to reduce HF hospitalization	Strong HFpEF RCT evidence, but not phenotype-specific	EMPEROR-Preserved, DELIVER, PRESERVED-HF	Hypertensive HFpEF not tested as a dedicated subgroup trial	Trials assessing SGLT2i sequencing with antihypertensive therapy
MRAs	May be considered in selected HFpEF patients	Limited and mixed evidence	TOPCAT, PATHWAY-2	Unclear benefit in hypertensive HFpEF with LVH/fibrosis	Trials targeting fibrosis/LVH phenotypes with careful renal and potassium monitoring
ARNI/RAAS inhibition	Used for BP control and selected HFpEF patients	Modest/inconclusive HFpEF outcome evidence	PARAGON-HF, hypertension trials	Uncertain role beyond BP lowering in hypertensive HFpEF	Phenotype-guided studies evaluating remodeling and clinical outcomes
Biomarker-guided management	Biomarkers recommended mainly for diagnosis/risk stratification	No dedicated biomarker-guided hypertensive HFpEF RCTs	NT-proBNP studies, sST2 observational data	No validated biomarker-driven treatment algorithm	Trials using NT-proBNP, sST2, troponin, and imaging to guide treatment intensity
Device-based therapies	Considered mainly for resistant hypertension or selected device indications	No dedicated hypertensive HFpEF outcome RCTs	SPYRAL HTN-ON MED, RDN studies, registry data	Effects on HFpEF outcomes, LVH regression, and symptoms remain uncertain	RCTs of renal denervation/BAT in resistant hypertensive HFpEF
Real-world implementation	Guidelines recommend individualized care in elderly/comorbid patients	Limited pragmatic HFpEF-specific evidence	Registries, frailty/adherence studies	Frailty, polypharmacy, CKD, and adherence underrepresented in RCTs	Pragmatic European registries and implementation trials in elderly multimorbid HFpEF

Abbreviations: HFpEF, heart failure with preserved ejection fraction; BP, blood pressure; CV, cardiovascular; RCT, randomized controlled trial; ESC, European Society of Cardiology; ESH, European Society of Hypertension; ACC, American College of Cardiology; AHA, American Heart Association; SGLT2, sodium–glucose cotransporter-2; MRA, mineralocorticoid receptor antagonist; LVH, left ventricular hypertrophy; ARNI, angiotensin receptor/neprilysin inhibitors; RAAS, renin–angiotensin–aldosterone system; NT-proBNP, N-terminal pro-B-type natriuretic peptide; ST2, soluble ST2; BAT, baroreflex activation therapy.

## Data Availability

No new data were created or analyzed in this study. Data sharing is not applicable to this article.
